# PW02-017 - New tools for the ISSAID society website

**DOI:** 10.1186/1546-0096-11-S1-A157

**Published:** 2013-11-08

**Authors:** I Touitou, F Milhavet

**Affiliations:** 1 Laboratoire de génétique des maladies rares et auto-inflammatoires, CHRU Montpellier, Montpellier Cedex 5, France

## Introduction

The **ISSAID** (International Society for Systemic Auto-Inflammatory Diseases) born in November 2005, is supported by a website portal.

## Objectives

The purpose of the website is to:

• gather resources related to the systemic auto-inflammatory diseases in order to facilitate contacts between interested physicians and researchers.

• provide support to share and rapidly disseminate information, thoughts, feelings and experiences to improve the quality of life of patients and families affected by systemic auto-inflammatory diseases, and promote advances in the search for causes and cures.

## Methods

Several existing items have been improved to provide extensive. Others have been developed. As decided at the last **ISSAID** council meeting in Amsterdam, access to specific sections of the **ISSAID** website is subjected to the payment of annual membership dues.

## Results

### Improved items

1. basic information

2. society missions and membership

3. previous and future meetings content

4. mutation and patient registries

5. image library showing characteristic physical features from patients suffering from AID

6. information related to laboratories such as quality control schemes for molecular diagnosis

7. useful links

### New items

We recently implemented a new "**expert views**" area with the possibility to

1. share professional experience

2. request expert advice

3. read updates on a specific topic, and research highlights

4. call for collaboration

## Conclusion

We do believe that this relooked **ISSAID** website will further help disseminate information, promote advances in the search for the causes of AID, and improve the quality of life and cure of patients affected by AID.

## Disclosure of interest

None declared.

